# Hollow screw fixation of type II avulsion fractures of the calcaneal tuberosity using the finite element analysis

**DOI:** 10.1097/MD.0000000000033816

**Published:** 2023-05-17

**Authors:** Chengwei Wang

**Affiliations:** a Department of Orthopedics, Huangshi Central Hospital, Affiliated Hospital of Hubei Polytechnic University, Huangshi, Hubei, China.

**Keywords:** avulsion fractures, biomechanics, calcaneal tuberosity, cannulated screws, finite element analysis

## Abstract

We aimed to establish a model of type II avulsion fractures of the calcaneal tuberosity treated with 2 hollow screws implanted in different directions and to analyze the biomechanical properties of the model using the finite element method. The Dicom data of the calcaneal bone obtained after computed tomography scan were inputted into Mimics 21.0 software and Geomagic Studio software to generate a 3D finite element digital model of the calcaneal bone. The model was then imported into SOLIDWORKS 2020 software. Based on the Beavis theory, the calcaneal bone was cut to build a type II avulsion fracture model of the calcaneal tuberosity; the calcaneal fracture was then simulated by internal fixation using hollow screws. Two screws were used to fix the calcaneal bone from the calcaneal tuberosity in different ways, resulting in 3 different calcaneal models (Model 1 involved 2 screws for fixing the fracture vertically; Model 2 had 2 screws for fixing the fracture cross-wise; and Model 3 had 2 screws for fixing the fracture parallelly). Three internal fixation models were loaded under the same conditions, and lines finite element analysis was then performed to calculate the stress distribution of the generated internal fixation models. Under the same loading conditions, compared with Models 2 and 3, Model 1 exhibited smaller maximum displacement values of the heel bone, maximum equivalent force values of the screws, and more dispersed stresses. Avulsion fractures of the calcaneal tuberosity can be treated using 2 screws to fix the fracture vertically (Model 1), which is more biomechanically relevant.

## 1. Introduction

Avulsion fractures of the calcaneal tuberosity are relatively rare, accounting for 1.3 to 2.7% of all calcaneal fracture cases.^[1,2]^ Advanced age, diabetes, and osteoporosis increase the risk of nodal avulsion.^[3]^ The incidence of avulsion fractures is the highest among women in their 60s.^[4]^ Although nonsurgical treatment is recommended for fractures with a displacement of <1 cm,^[5]^ surgical treatment is recommended for larger fractures to restore the functions of the gastrocnemius–fibularis muscle complex.^[6]^ However, the avulsed fragments are usually small and the calcaneal bone is osteoporotic, together with the skin complications, making its treatment more and more difficult.

Lag screw fixation, tension band wiring, suture anchors, and k-wire are the common techniques used to repair calcaneal tuberosity fractures. However, the optimal surgical technique remains a subject of debate as the available techniques continue to evolve.^[6–8]^ Moreover, there is no theoretical basis for deciding which fixation method is more biomechanically relevant. Finite element analysis (FEM) is a method that is used to analyze dynamic and static physical systems.^[9]^ It is now considered an effective computational experimental method in the field of orthopedic biomechanics.

Based on the above theory, we first developed a 3D finite element model of a type II heel tuberosity fracture and then mechanically loaded the model with different internal screw fixation methods for biomechanical optimization. The primary objectives of this study were as follows: to analyze the stresses under different screw fixation methods using the same loading conditions and to investigate the biomechanics of the heel fracture fixation method and to determine the optimal solution for the screw fixation of type II heel tuberosity fractures through biomechanical comparisons.

## 2. Materials and methods

### 2.1. General data

A 28-year-old healthy male volunteer (height, 175 cm; weight, 75 kg) was recruited as the study participant. His X-ray examination showed no deformity or injury in his foot. This study was approved by the Ethics Committee of Huangshi Central Hospital, China.

### 2.2. Equipment and software

We used the following software in the study: SOMATOM 64-row computed tomography (CT) (Siemens Ltd., Germany); Mimics 21.0 medical imaging software (Materialise Ltd., Belgium); Geomagic Studio 3D optimization software (Raindrop Ltd., USA); SOLIDWORKS 2020 (Dassault Systems Ltd., USA); and ANSYS 17.0 FEM software (ANSYS Ltd., USA).

### 2.3. Data collection

Under the SOMATOM 64-row CT, the volunteer was scanned from the middle segment of the tibia with his foot positioned in the neutral position. The slice thickness was maintained at 0.625 mm, and 360 projection CT images were obtained to generate a 512 × 512 matrix (digital imaging and communications in medicine format).

### 2.4. Establishment of the 3D model

**Figure 1.**
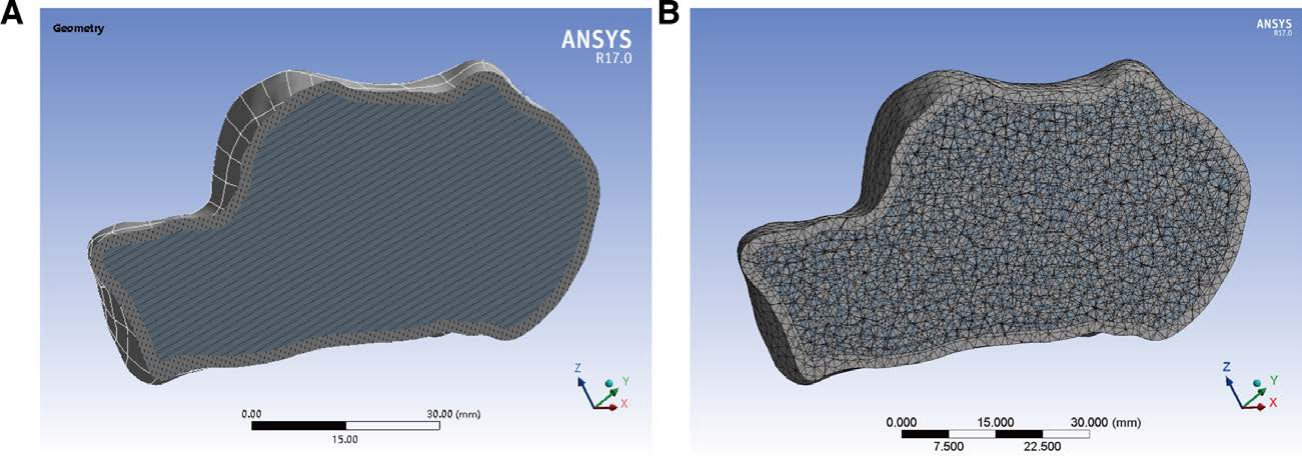
Calcaneal bone finite element 3D model. (A) Cortical and cancellous bone section of calcaneal bone. (B) Calcaneal bone mesh section.

Mimics 21.0 software can directly read the digital imaging and communications in medicine format files to create a 3D calcaneus model (Fig. 1A). The model was then imported into Geomagic Studio 2013, and the surface mesh was optimized by surface smoothing and triangle reduction based on the 3D solid model (Fig. 1B). The 3D model was read using SOLIDWORKS 2020, and the calcaneus model was then cut according to the results of a calcaneal fracture classification to create a model of type II avulsion fractures of the calcaneal tuberosity.

Based on the screw information provided by the equipment dealer, we positioned 2 cannulated screws with a 3.5-mm diameter; the geometric parameters of the screws were loaded into SOLIDWORKS 2020, and 3 internal fixation models were created based on the experimental design. Model 1 involved 2 screws for fixing the fracture vertically; Model 2 had 2 screws for fixing the fracture crosswise; and Model 3 had 2 screws for fixing the fracture parallelly (Fig. 3).

**Figure 2.**
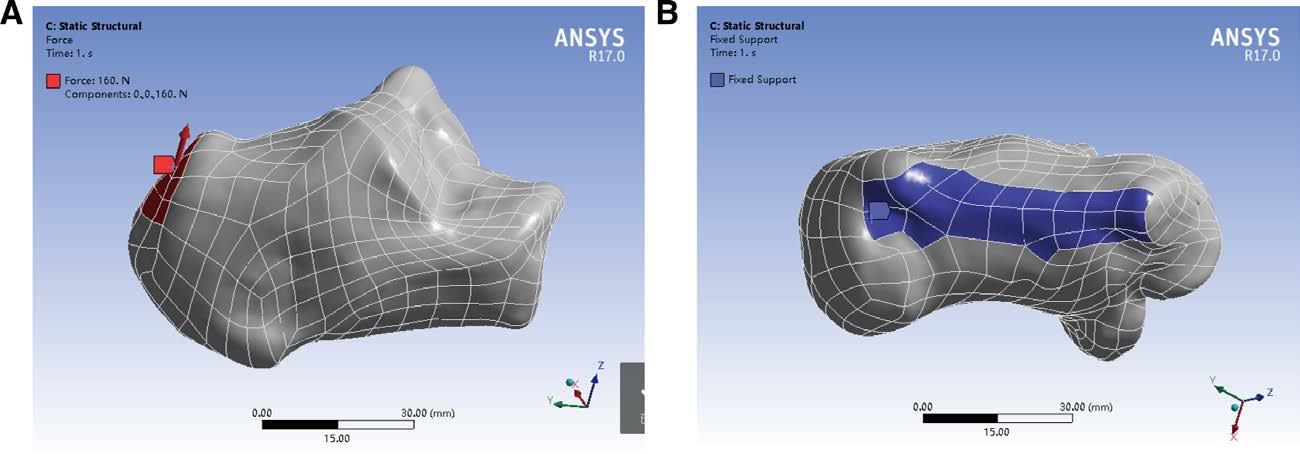
Calcaneal bone finite element 3D model. (A) Load map of calcaneal bone (the red arrow indicates the direction of the force). (B) Constraint at the base of the calcaneal bone (blue indicates the constraint range).

The calcaneus model was imported into ANSYS 17.0 software in 3 different fixation states, and a 3D finite element numerical model of the calcaneus was generated by setting the unit elements, assigning material properties, and dividing the cell mesh. All the tissues involved in the model were simplified to anisotropic homogeneous elastic material states; the heel bone thickness for the cortical bone was set to 2 mm, and the inner part comprised homogeneous cancellous bone. The cortical calcaneus thickness was set at 1 mm, and the inner part comprised homogeneous cancellous bone. The performance parameters of the various materials within the calcaneus model are presented in Table 1.^[10]^

**Table 1:** Elastic property parameter of internal materials of calcaneal model.

Materials	Cortical bone	Cancellous bone	Cannulated screw
Modulus of elasticity (MPa)	7300	100	110000
Poisson ratio	0.3	0.3	0.3

### 2.5. External force loading

The loading point of the calcaneus model was set at the calcaneus tuberosity, which was loaded bottom upwards (160 N); the pulling force on the calcaneus generated by the intrinsic foot muscles of the calcaneus could be counteracted by the principles of force synthesis and decomposition (Fig. 2A). The joint surface of the calcaneus dice and the lowest point of contact between the calcaneus and the ground were considered as restraint points (Fig. 2B). The forces on the heel bone in the neutral position were simulated using the 3D calcaneus model, as shown in Figure 1.

## 3. Results

Given the results of the FEM, the maximum values were found to be more relevant for the stress–strain situation after force loading. Therefore, the maximum values of the overall equivalent stress of the screw (Fig. 5) and the calcaneus displacement (Fig. 4) were recorded as the test measures in this study (Table 2).

**Table 2:** Stress and strain condition of different material of different calcaneal of models loading.

Group	Maximum calcaneal displacement (mm)	Maximum overall equivalent stress of cannulated screw (MPa)
Model 1	0.46410	186.78
Model 2	0.99696	454.56
Model 3	0.36278	329.98

**Figure 3.**
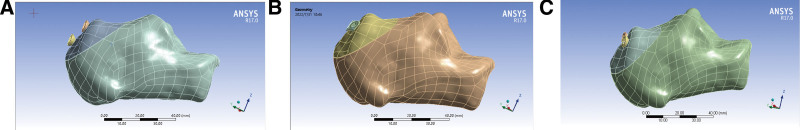
Model obtained after performing different screw internal fixation methods following a type 2 fracture of the calcaneal tuberosity. (A) Model 1: 2 screws for fixing the fracture vertically; (B) Model 2: 2 screws for fixing the fracture crosswise; and (C) Model 3: 2 screws for fixing the fracture parallelly.

**Figure 4.**
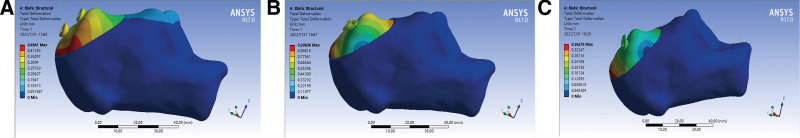
Calcaneus displacement. (A) Model 1: the maximum displacement was 0.4641 mm, mainly noted at the posterior lateral aspect of the calcaneus tuberosity; (B) Model 2: the maximum displacement was 0.99696 mm, mainly concentrated at the posterior medial aspect of the calcaneus tuberosity; and (C) Model 3: the maximum displacement was 0.36278 mm, which was concentrated just posterior to the calcaneus tuberosity.

**Figure 5.**
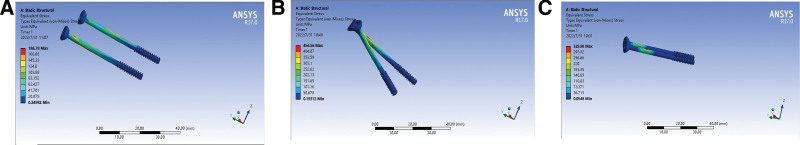
Stress distribution in the counteracted screws. (A) Model 1: the maximum stress was 186.78 MPa, which was mainly concentrated at the proximal end of the lower counteracted screw; (B) Model 2: the maximum stress was 454.56 MPa, which was mainly concentrated at the proximal end of the upper counteracted screw; and (C) Model 3: the maximum stress was 329.98 MPa, which was mainly concentrated at the proximal end of the outer counteracted screw.

### 3.1. Calcaneus displacement

After loading, the minimum and maximum calcaneus displacement values in the posterior lateral aspect of the calcaneus tuberosity in Model 1 were 0 mm and 0.4641 mm, respectively. The minimum and maximum displacement values in the posterior medial aspect of the calcaneus tuberosity in Model 2 were 0 mm and 0.99696 mm, respectively. The minimum and maximum calcaneus displacement values just posterior to the calcaneus tuberosity in Model 3 were 0 mm and 0.36278 mm, respectively (Fig. 4).

### 3.2. Stress distribution in the counteracted screws

After loading, the maximum stress in Model 1 was 186.78 MPa, and the stress was mainly concentrated at the proximal end of the lower counteracted screw. The maximum stress in Model 2 was 454.56 MPa, mainly concentrated at the proximal end of the upper counteracted screw. The maximum stress in Model 3 was 329.98 MPa, mainly concentrated at the proximal end of the outer counteracted screw. The overall maximum stress of the counteracted screws was 453.56 MPa, mainly concentrated at the proximal end of the counteracted screws, and the maximum calcaneus displacement value was 0.99696 mm in model 2.

## 4. Discussion

Beavis^[11]^ developed a classification system that includes 3 types of avulsion fractures of the calcaneus tuberosity: type I is a cuff fracture wherein the cortical shell is avulsed from the posterior tuberosity; type II is a rostral fracture wherein an angled fracture line extends posteriorly from the uppermost part of the posterior tuberosity; and type III is a sub-bursal avulsion fracture occurring from the middle third of the posterior tuberosity.

The force exerted by the Achilles tendon is a major factor to be considered in the surgical treatment of avulsion fractures of the calcaneus tuberosity. Herein, we aimed to examine this particular avulsion fracture in light of what is already known about avulsion fractures in other parts of the body.^[12]^

Nodal Achilles fractures commonly occur in patients with diabetes and osteoporosis; the poor bone quality in these patients increases the likelihood of failure of cancellous bone screw fixation and bone healing.^[13]^ Moreover, Ramaswamy^[14]^ reported that the clamping ability of screws is directly proportional to bone density.

Hollow nails, cancellous bone screws, and cortical bone screws are the commonly used screws in the treatment of heel tuberosity fractures.^[5]^ In this study, we used hollow nails and recorded their stresses and displacements.^[15]^ The maximum displacement value was 0.99696 mm, concentrated at the posterior medial aspect of the calcaneus tuberosity, whereas the maximum stress was 454.56 MPa, mainly concentrated at the proximal end of the upper counteracted screw in model 2. Hollow screws should be able to resist stress adequately and compress the fracture ends to facilitate healing.^[5,16]^

In case of avulsion fractures of the heel, the skin behind the heel must be promptly evaluated.^[7,17]^ If skin tenting is present, the fracture must be immediately repositioned and fixed to avoid the risk of skin necrosis. Hollow nails are minimally invasive, offer easy fixation, and cause limited skin irritation and fewer skin necrosis complications; these properties make them an ideal choice for fracture treatment.^[18]^

Our study has some limitations. First, the calcaneus is complex, and the trabeculae within the cancellous bone are not homogeneous; however, the inner part of the calcaneus is inevitably set as homogeneous for performing loading calculations as required. Second, the mechanical influence of the tendinous tissues surrounding the calcaneus, such as the Achilles tendon, metatarsal fascia, ligaments, and the toe flexor tendon, on the heel bone is yet to be studied in a large number of biomechanical studies.^[19]^

## 5. Conclusions

We developed a type 2 fracture model of the heel tuberosity based on the findings of Beavis theory.^[11]^ We used 2 hollow nails to fix the fracture ends of the heel tuberosity in different ways. After FEM analysis, we found that it is more biomechanical to fix the fracture end vertically with 2 screws (Model 1). This approach is biomechanically relevant and will provide guidance to orthopedic surgeons wishing to perform this procedure in the clinical setting.

## Author contributions

**Conceptualization:** Chengwei Wang.

**Data curation:** Chengwei Wang.

**Formal analysis:** Chengwei Wang.

**Investigation:** Chengwei Wang.

**Methodology:** Chengwei Wang.

**Writing – original draft:** Chengwei Wang.

**Writing – review & editing:** Chengwei Wang.
